# Bioactive Cellulose Nanocrystal-Poly(ε-Caprolactone) Nanocomposites for Bone Tissue Engineering Applications

**DOI:** 10.3389/fbioe.2021.605924

**Published:** 2021-02-25

**Authors:** Jung Ki Hong, Shelley L. Cooke, Abby R. Whittington, Maren Roman

**Affiliations:** ^1^Macromolecules Innovation Institute, Virginia Tech, Blacksburg, VA, United States; ^2^Department of Materials Science and Engineering, Virginia Tech, Blacksburg, VA, United States; ^3^Department of Chemical Engineering, Virginia Tech, Blacksburg, VA, United States; ^4^Department of Sustainable Biomaterials, Virginia Tech, Blacksburg, VA, United States

**Keywords:** cellulose nanocrystal, poly(ε-caprolactone), nanocomposite, biomineralization, bone scaffold

## Abstract

3D-printed bone scaffolds hold great promise for the individualized treatment of critical-size bone defects. Among the resorbable polymers available for use as 3D-printable scaffold materials, poly(ε-caprolactone) (PCL) has many benefits. However, its relatively low stiffness and lack of bioactivity limit its use in load-bearing bone scaffolds. This study tests the hypothesis that surface-oxidized cellulose nanocrystals (SO-CNCs), decorated with carboxyl groups, can act as multi-functional scaffold additives that (1) improve the mechanical properties of PCL and (2) induce biomineral formation upon PCL resorption. To this end, an *in vitro* biomineralization study was performed to assess the ability of SO-CNCs to induce the formation of calcium phosphate minerals. In addition, PCL nanocomposites containing different amounts of SO-CNCs (1, 2, 3, 5, and 10 wt%) were prepared using melt compounding extrusion and characterized in terms of Young's modulus, ultimate tensile strength, crystallinity, thermal transitions, and water contact angle. Neither sulfuric acid-hydrolyzed CNCs (SH-CNCs) nor SO-CNCs were toxic to MC3T3 preosteoblasts during a 24 h exposure at concentrations ranging from 0.25 to 3.0 mg/mL. SO-CNCs were more effective at inducing mineral formation than SH-CNCs in simulated body fluid (1x). An SO-CNC content of 10 wt% in the PCL matrix caused a more than 2-fold increase in Young's modulus (stiffness) and a more than 60% increase in ultimate tensile strength. The matrix glass transition and melting temperatures were not affected by the SO-CNCs but the crystallization temperature increased by about 5.5°C upon addition of 10 wt% SO-CNCs, the matrix crystallinity decreased from about 43 to about 40%, and the water contact angle decreased from 87 to 82.6°. The abilities of SO-CNCs to induce calcium phosphate mineral formation and increase the Young's modulus of PCL render them attractive for applications as multi-functional nanoscale additives in PCL-based bone scaffolds.

## Introduction

Bone is one of a few organs in the body that have the ability to self-regenerate without scar tissue formation following injury. However, the self-healing capacity of bone is limited to smaller than critical-sized bone defects. Critical-sized defects typically need to be repaired with bone grafts, such as autografts, allografts, or xenografts, or with metal or ceramic implants. These approaches have several limitations including donor site morbidity, donor bone supply shortage, infection, corrosion, stress shielding, and secondary surgery.

Compared to ceramics and metals, bioresorbable polymers have several inherent benefits for bone scaffold applications, including 3D printability and biodegradability. However, their inferior strength, stiffness, and bioactivity present critical challenges that need to be addressed. A mechanical mismatch between the scaffold and surrounding bone tissue could result in bone resorption through a stress shielding effect or failure of the scaffolds at the defect sites (Liu et al., 2006; Sultana, 2013).

Bone is composed to 65% of mineral and 35% of an organic matrix. The mineral phase, primarily comprising hydroxyapatite (HA), Ca_10_(PO_4_)_6_(OH)_2_, plays important roles in both the mechanical strength of bone and mineral ion homeostasis. The surface layer of bone, called cortical bone, is dense (~10% porosity) and provides most of the supportive and protective function of the skeletal system (Sikavitsas et al., 2001). Cortical bone has a tensile strength of 89–151 MPa and a Young's modulus of 6–17 GPa (Athanasiou et al., 2000). Cancellous bone, on the other hand, making up 20% of the total bone mass of the skeleton and found in the bone interior, has a sponge-like morphology (50–90% porosity) and mechanical properties up to 10 times inferior to those of cortical bone. Nevertheless, the mechanical properties of cancellous bone are as challenging to match as those of cortical bone when aiming for comparable scaffold porosity.

A polymer that has attracted considerable attention for bone scaffold applications is poly(ε-caprolactone) (PCL) (Porter et al., 2009; Ruckh et al., 2010; Perez et al., 2012). Among its advantages are a relatively low melting temperature, the ability to be slowly bioresorbed upon implantation, and its status with the U.S. Food and Drug Administration as approved for use in medical devices. The main drawbacks of PCL are its insufficient load-bearing properties and its lack of bioactivity in regards to biomineralization (i.e., HA formation).

Several studies have reported improvements in the properties of PCL-based bone scaffolds upon incorporation of nanoscale additives, including HA nanocrystals (Wutticharoenmongkol et al., 2006; Jing et al., 2015), nanosized calcium silicate (Wei et al., 2009), or carbon nanotubes (Pan et al., 2012). Observed improvements include enhanced mechanical properties (Wutticharoenmongkol et al., 2006; Pan et al., 2012; Jing et al., 2015) and superior biomineralization (Wei et al., 2009; Jing et al., 2015). The nucleation/growth of biominerals on surfaces has been shown to strongly depend on the chemical properties of the surface, impacting the supersaturation of the growth environment (Sato et al., 2001; Weiner and Dove, 2003; Colfen, 2010; Dey et al., 2010). Tanahashi and Matsuda (1997) examined the rate of apatite formation on self-assembled monolayers (SAMs) with different terminal functional groups in simulated body fluid (SBF). The most potent functional group for apatite formation was the negatively charged -PO_4_H_2_ followed by the -COOH group. Non-ionic groups, such as -CONH_2_ and -OH, and a positively charged group (NH_2_) showed weaker nucleating ability, and it was found that apatite formation was inhibited on CH_3_-terminated SAMs (Tanahashi and Matsuda, 1997).

This study tests the hypothesis that cellulose nanocrystals (CNCs), functionalized through surface oxidation, can act as multi-functional PCL scaffold additives that (1) improve the mechanical properties of PCL and (2) induce biomineral formation upon PCL resorption. CNCs are elongated nanoparticles, derived from various cellulose sources, including wood, plant, tunicate, algae, or bacterial cellulose (Moon et al., 2011), that have garnered interest for a number of potential applications because of their unique characteristics, including excellent mechanical properties, reactive surface chemistry, biodegradability, biocompatibility, low ecotoxicological risk, and comparatively low cost (Fleming et al., 2000; Kovacs et al., 2010; Lin et al., 2012; Domingues et al., 2014). Over the past three decades, cellulose nanocrystals (CNCs) have drawn significant interest as nanofillers in polymer matrices (Dufresne, 2008; Habibi and Dufresne, 2008; Habibi et al., 2008; Chen et al., 2009). The specific objectives of this study were to determine whether surface oxidation of CNCs, resulting in the conversion of hydroxymethyl- to carboxyl groups, enhances their ability to induce biomineralization and whether surface-oxidized CNCs (SO-CNCs) improve the mechanical properties of PCL.

## Materials and Methods

### Preparation of Sulfuric Acid-Hydrolyzed CNCs (SH-CNCs)

SH-CNCs were prepared from bleached, dissolving-grade softwood sulfite pulp (Temalpa 93A), kindly provided by Tembec, Inc. (Témiscaming, QC, CA). Milled (60 mesh, Thomas Wiley® Mini-Mill, Thomas Scientific) pulp was hydrolyzed for 60 min with 64 wt% sulfuric acid (96.2 wt%, Fischer Scientific) at 45.5°C and an acid-to-pulp ratio of 10 mL/g. After 60 min, the reaction mixture was diluted by addition of 2.5 L cold (~4°C), deionized (DI) water (18.2 MΩ·cm, Millipore Direct-Q5 Ultrapure Water System) and the acid was removed by centrifugation at 4,500 rpm for 20 min at 4°C (IEC CENTRA GP8R Refrigerated Centrifuge, Thermo Electron Corporation) followed by discarding of the supernatant. The sediment was redispersed in DI water and centrifuged. This step was repeated three times. Then, the collected sediment was transferred to dialysis tubing (Spectra/Por 4, molecular weight cut off of 12–14 kDa) and dialyzed against DI water, exchanged daily, for 1 week to remove residual acid. After the dialysis, the SH-CNC suspension was sonicated (BRANSONIC® 3510) for 30 min and filtered through a poly(vinylidene fluoride) (PVDF) syringe filter (0.45 μm, Whatman, Ltd.). The final concentration of the obtained aqueous SH-CNC suspension was ~1 wt%.

### Preparation of Surface-Oxidized CNCs (SO-CNCs)

Surface oxidation of SH-CNCs (Supplementary Figure 1) was performed according to the method of Araki et al. (2001) with minor modifications, based on the method of Habibi et al. (2006). Briefly, 500 g of 1 wt% aqueous SH-CNC suspension, 0.5 g of (2,2,6,6-tetramethylpiperidin-1-yl)oxyl (TEMPO, free radical, 98%, Sigma-Aldrich), and 5 g of sodium bromide (99+%, extra pure, anhydrous, Acros Organics) were thoroughly mixed in a 1 L flask with a magnetic stir bar at room temperature. The reaction was started by gradual addition of 50 mL sodium hypochlorite solution (reagent grade, available chlorine 10–15%, Sigma-Aldrich) so that the pH of the reaction mixture remained in the range 10.2–10.5. The reaction was continued for 3 h under addition of sodium hydroxide (0.5 M) to maintain the pH. The reaction was quenched by addition of 5 mL of methanol and the mixture was transferred to dialysis tubing and dialyzed against DI water (refreshed daily) for 2 weeks. Finally, the obtained aqueous suspension of SO-CNCs was sonicated for 30 min and filtered through a 0.45 μm PVDF syringe filter. The concentration of both suspensions (SH-CNC and SO-CNC) was adjusted to 3 wt% with a rotary evaporator (Büchi Rotavapor R-200) using a water bath temperature of 40°C. The stock suspensions were stored in a refrigerator until used.

### Conductometric Titration

The surface charge densities of SH-CNCs and SO-CNCs were determined by conductometric titration. The aqueous suspensions were placed over a small amount of ion-exchange resin (Rexyn®I-300 (H-OH), certified, research grade, Fisher Scientific) for 12 h and filtered through 1.0 μm PVDF syringe filters before the titration. The ion exchange resin-treated SH-CNCs and SO-CNCs are denoted SH-CNC-I and SO-CNC-I, respectively. Titrations of 45 mL of 0.20 wt% SH-CNC suspension were carried out after addition of 5 mL of 0.01 M potassium chloride to increase the ionic strength. Titrations of 40 mL of 0.20 wt% SO-CNC suspension were conducted after addition of 20 mL of 0.01 M hydrochloric acid. The electrical conductivity was recorded with a pH/conductivity meter (Mettler Toledo SevenMulti S47 pH/conductivity meter with an InLab 730 conductivity probe) every 30 s after addition of 100 μL aliquots of 0.01 M sodium hydroxide by micropipette. Reported values are means of duplicate measurements. The surface charge density (σ) was calculated with the following equation (Jiang et al., 2010):

$$\begin{array}{l} \sigma =\frac{C_{NaOH}\times V_{NaOH}}{C_{CNC}\times \alpha_{CNC}} \end{array}$$

where *C*_NaOH_ is the concentration of NaOH, *V*_NaOH_ is the volume of NaOH at the equivalence point, *C*_CNC_ is the concentration of the CNC suspension, α_CNC_ is the amount of CNC suspension titrated. For the SO-CNCs, *V*_NaOH_ was defined as *V*_2_-*V*_1_, where *V*_1_ (1st equivalence point) is the volume of NaOH needed to neutralize excess HCl and the surface sulfate groups and *V*_2_ (2nd equivalence point) is the volume of NaOH needed to neutralize the carboxyl groups, respectively.

### Biomineralization of SH-CNCs and SO-CNCs *in vitro*

An *in vitro* biomineralization study was performed in SBF at 37°C. SBF with nearly identical ion concentrations to those of human blood plasma was carefully prepared as described in Kokubo and Takadama (2006), henceforth denoted corrected SBF (c-SBF). For CNC mineralization, 100 mL aliquots of 1 wt% aqueous suspensions of either SH-CNCs or SO-CNCs were placed in dialysis tubing and suspended in 3 L of SBF at 37°C for up to 800 h under stirring. The SBF was exchanged every 24 h. Samples of the mineralized CNCs (10 mL aliquots) were collected at different incubation time points and dialyzed against DI water for 48 h. The aqueous suspensions of mineralized SH-CNCs and SO-CNCs were stored at 4°C prior to analysis.

### Inductively Coupled Plasma Atomic Emission Spectroscopy (ICP-AES)

ICP-AES (Spectro ARCOS, Spectro Analytical Instruments, Inc.) was used to analyze the concentrations of elements such as sulfur, phosphorus, and calcium before and after mineralization of the CNCs. Five milliliter of CNC suspensions (5 mg/mL) were treated with 25 mL of nitric acid (70%, TraceMetal Grade, Fisher Scientific) for 2 h at 60°C in an ultrasonic bath (40 kHz, 130 W). One milliliter of the thus digested suspension was added to 39 mL of DI water resulting in a final CNC concentration of 0.02 mg/mL in HNO_3_. Reported values are means of three measurements.

### SO-CNC/PCL Nanocomposites Fabrication

The SO-CNCs were suspended in acetonitrile (HPLC Grade, Fisher Scientific) by solvent exchange. The solvent exchange process was performed by addition of acetonitrile to the aqueous SO-CNC suspension. The mixture was centrifuged at 5,000 rpm (SORVALL LEGEND X1R Centrifuge, Thermo Scientific) for 20 min and fresh acetonitrile was added to the collected sediment. This step was followed by homogenization (Power Gen 500, Sawtooth 10 x 95 mm, Fisher Scientific) for 5 min and sonication for 30 min. This process was repeated three times. PCL (Mw = 70,000–90,000, Sigma Aldrich) was dissolved in dichloromethane (Fisher Scientific). The ratio of PCL to dichloromethane was 1:10 (w/v). The solvent-exchanged SO-CNC suspension was sonicated at 40°C for 30 min under stirring (300 rpm) and the PCL solution was slowly added before solvent casting into a glass petri dish. The cast film was allowed to dry at room temperature for 48 h, then placed in a vacuum oven at 40°C for 24 h. The solvent cast films were cut into small pellets, then thoroughly rinsed with DI water and dried in a vacuum oven at 40°C for 24 h. Finally, the pellets were extruded into a 3 mm (diameter) filament with a twin-screw extruder (HAAKE MiniLab II, ThermoFisher Scientific) at 80°C. SO-CNC/PCL nanocomposites were prepared with SO-CNC contents of 1, 2, 3, 5, and 10 wt%.

### Atomic Force Microscopy (AFM)

AFM samples of SH-CNCs and SO-CNCs were prepared from 0.002 wt% aqueous suspensions. After 10 min sonication, 15 μL of each CNC suspension was spin coated at 4,000 rpm for 1 min with a spin coater (Laurell model WS-400B-6NPP/LITE) onto a freshly cleaved mica disc (diameter: 0.5 in., Ted Pella) mounted with epoxy adhesive resin onto a standard microscope slide. The spin coated CNC samples were dried overnight at 60°C under vacuum. AFM samples of the PCL nanocomposites were prepared by immersion of nanocomposite filaments in liquid N_2_ followed by fracture of the filament in flow direction of the melt compounding extrusion process. Fresh fracture surfaces were imaged under ambient conditions in intermittent contact (AC) mode with an Asylum Research MFP3D-Bio atomic force microscope using standard silicon probes (Olympus OMCL-AC160TS, resonant frequency: ~300 kHz, spring constant: ~42 N/m, nominal tip radius: <10 nm). Images were recorded with a resolution of 512 points/scan and analyzed using IGOR pro software (RRID:SCR_000325) using identical parameters for the mask tool, flattening tool, z-scale, and phase range.

### Cytotoxicity of SH-CNCs and SO-CNCs

Mouse preosteoblast (MC3T3, ATCC) cells were cultured in alpha minimum essential medium (α-MEM, Life Technologies) with 10% fetal bovine serum (FBS, Life Technologies) and 1% penicillin-streptomycin (Life Technologies). Aliquots of the SH-CNC and SO-CNC stock suspensions (30 mg/mL) were diluted with DI water to yield samples with four different concentrations: 1.67, 3.33, 6.67, and 20.00 mg/mL. The samples were filtered through 0.22 μm PVDF syringe filters and exposed to ultraviolet light for sterilization. One hundred fifty microliters of each sample was added to 850 μL of α-MEM containing 10,000 MC3T3 cells for cytotoxicity testing. After 24 h, 20 μL of trypan blue assay solution (0.4%, Life Technologies) was added. Live cells (clear) and dead cells (blue) were counted with an optical microscope (Fisher Scientific) and averaged with a hemacytometer. Experiments were carried out in triplicate and referenced against DI water (150 μL).

### Tensile Tests

Tensile tests of SO-CNC/PCL nanocomposite filaments were performed with an MTS Sintech 10/GL Material Testing Workstation equipped with a 100 N load cell. A cross head speed of 10 mm/min was used at ambient conditions (in air at room temperature) was used. Reported values are means of quintuplicate measurements. Measurements with yield near the grips were excluded.

### Thermal Analysis: DSC

DSC measurements were performed with a TA Instruments Q2000 differential scanning calorimeter that had been calibrated with indium and sapphire standards. Nitrogen, at a flow rate of 50 mL/min, was used as the purge gas. ~5 mg of sample was placed in a standard aluminum DSC pan (TA Instrument). The DSC scans were done using a heating/cooling/heating/cooling protocol with a heating rate of 10°C/min and a temperature range of −75 to 100°C. Experiments were done in triplicate. The glass transition temperature (*T*_g_), melting temperature (*T*_m_), crystallization temperature (*T*_c_), and enthalpy of fusion, Δ*H*_*f*_, were measured with the TA Instruments' Universal Analysis 2000 software. The degree of matrix crystallinity, *X*_*c*_, was calculated from Δ*H*_*f*_ with the following equation (Runt, 1980):

$$\begin{array}{l} X_{c}=\frac{1}{\left(1-\omega_{f}\right)}\times \frac{\Delta H_{f_{sample}}}{\Delta H_{f_{PCL}}}\times 100\% \end{array}$$

where ω_*f*_ is the weight fraction of the filler in the composite and Δ*H*_*f*_*PCL*__ is the heat of fusion of the matrix polymer at 100% crystallinity. The percentage of crystallinity was estimated using a value of 139.5 J/g for the heat of fusion of 100% crystalline PCL (Pitt et al., 1981).

In addition, the thermal degradation temperatures of SO-CNC (film form), pure PCL, and PCL with 10 wt% SO-CNCs were determined by thermogravimetric analysis (TGA, TA Instruments TGA Q500) (Supplementary Figure 2). Approximately 10–15 mg of sample was placed into a platinum TGA sample pan. Thermogravimetric (TG) and derivative TG (DTG) curves between ~30 and 500°C were recorded with a heating rate of 10°C/min using air as sample purge gas.

### Contact Angle Measurements

For contact angle measurements, SO-CNC/PCL nanocomposite films (~5 mm in width, ~2 mm in thickness) were prepared using a twin-screw extruder (HAAKE MiniLab II, ThermoFisher Scientific). Measurements were performed with an FTA 200 Dynamic Contact Angle Analyzer equipped with a motor-driven syringe. Droplets of approximately 1 μl of each DI water and SBF were carefully deposited onto the sample surface using a 250 μl syringe (Hamilton gastight®) with a stainless steel needle. Images were recorded within 2 s and the contact angle was analyzed with Drop Shape Analysis software (FTA32 Video 2.1). Reported values are means of three measurements.

### Optical Light Microscopy

Thin slices of ~50 μm thickness were microtomed off of the transverse surface (perpendicular to the flow direction) of the PCL nanocomposites using a sliding microtome (Model 860, American Optical Company). The microtomed slices were placed on regular microscopy cover glasses and heated to 300°C for 10 min using the TGA. After that, optical microscopy images were recorded with a Canon EOS 20D digital single-lens reflex camera (8.2 megapixels) mounted onto a Zeiss Axioskop 40 A POL microscope.

## Results

**Figure 1:**
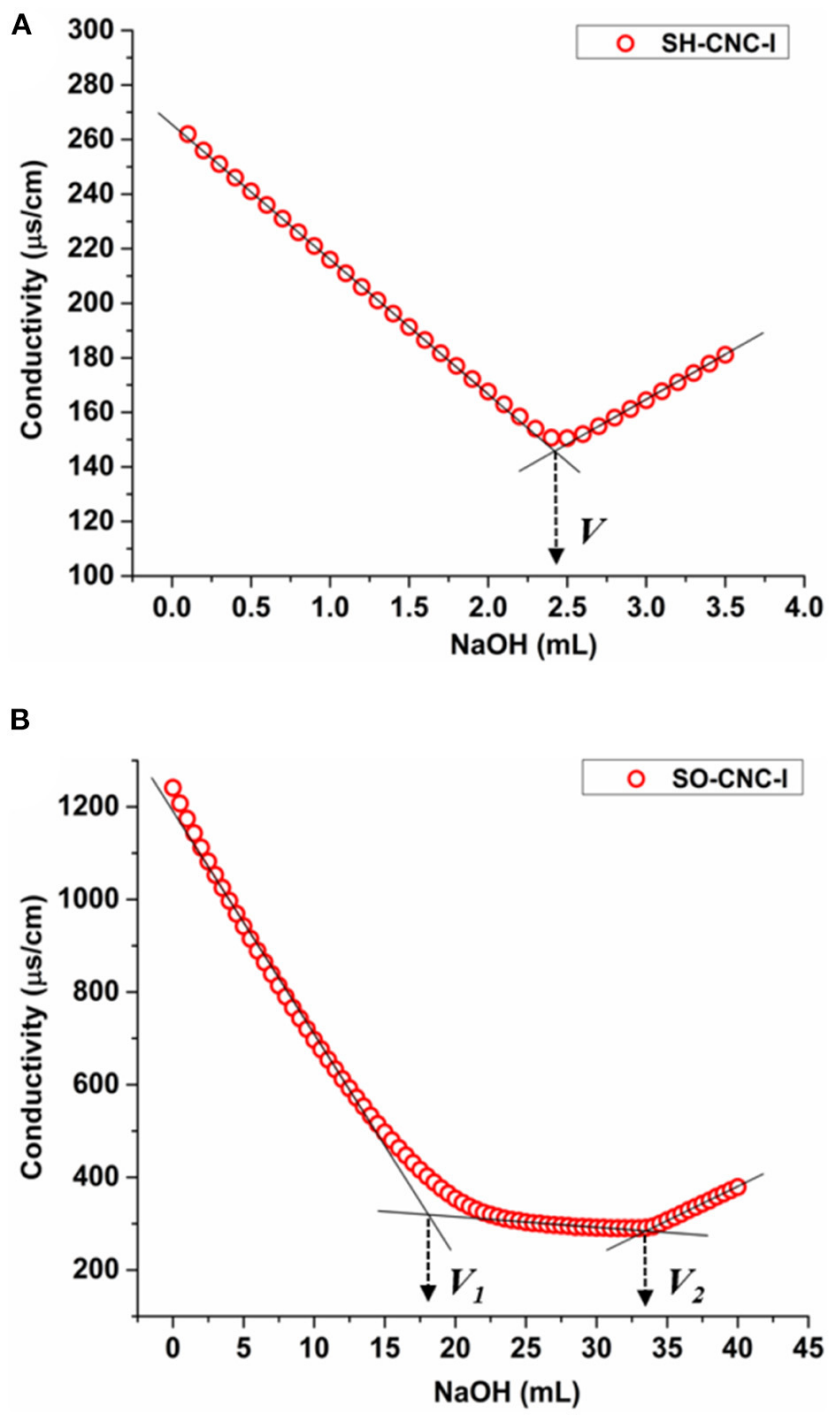
Conductometric titration curves for **(A)** SH-CNC-I and **(B)** SO-CNC-I.

SH-CNCs are known to carry sulfate groups on their surface, in addition to primary and secondary hydroxyl groups. To study whether the presence of carboxyl groups enhances the ability of CNCs to induce biomineralization, SH-CNCs were modified by TEMPO-mediated oxidation to give SO-CNCs. The reaction converts some of the primary hydroxyl groups on the CNC surface to carboxyl groups (Supplementary Figure 1). The presences of carboxyl groups was confirmed by FTIR spectroscopy (Supplementary Figure 3). The surface charge densities of SH-CNC and SO-CNC, after treatment with ion exchange resin (denoted as SH-CNC-I and SO-CNC-I, respectively) for removal of residual electrolyte, were determined by conductometric titration (Figure 1). The titration curve of SH-CNC-I (Figure 1A) showed an initial decrease of conductivity, corresponding to the neutralization of partially dissociated sulfate groups, followed by an increase upon further NaOH addition, due to an excess of titrant. The amount of sulfate groups per mass of SH-CNC-I is calculated from the volume of NaOH at the equivalence point (V). The sulfate group density of SH-CNC-I was calculated to be 0.271 ± 0.002 mmol/g (compared to 0.316 ± 0.007 mmol/g for SH-CNC, see Supplementary Figure 4 and Supplementary Table 1). Titration curves of SO-CNC-I, on the other hand (Figure 1B), exhibited two equivalence points. The first equivalence point (*V*_1_) corresponds to the neutralization of added HCl and any remaining sulfate groups. The second equivalence point (*V*_2_) corresponds to the neutralization of the carboxyl groups. The number of carboxyl groups per mass of SO-CNC-I was estimated from the volume of the titrant between the two equivalence points. The obtained surface charge density of SO-CNC-I was 1.997 ± 0.142 mmol/g (compared to 1.840 ± 0.004 mmol/g for SO-CNC, Supplementary Table 1). Thus, the surface charge density of SO-CNC-I was about 7 times (6 times before ion exchange treatment) higher than that of SH-CNC-I.

The sulfur content of the CNCs before and after ion exchange treatment was measured by ICP AES. Table 1 compares the sulfur contents of the CNC samples obtained by conductometric titration and ICP AES (see also Supplementary Table 1). As seen in the table, the values obtained by the two methods were in good agreement. Both, the surface oxidation and treatment with ion exchange resin, resulted in a decrease in the sulfur content of CNCs. According to the ICP AES data, surface oxidation caused a 21% reduction in the sulfur content (16% reduction after ion exchange resin treatment).

**Table 1:** Sulfur content of CNCs before and after treatment with ion exchange resin measured by conductometric titration and ICP analysis.

	**Sulfur content (mg/g)**	
**Sample**	**Conductometric titration**	**ICP AES analysis**
SH-CNC	10.11 ± 0.22	10.83 ± 0.20
SH-CNC-I	8.67 ± 0.05	9.00 ± 0.21
SO-CNC	–	8.56 ± 0.21
SO-CNC-I	–	7.59 ± 0.08

**Figure 2:**
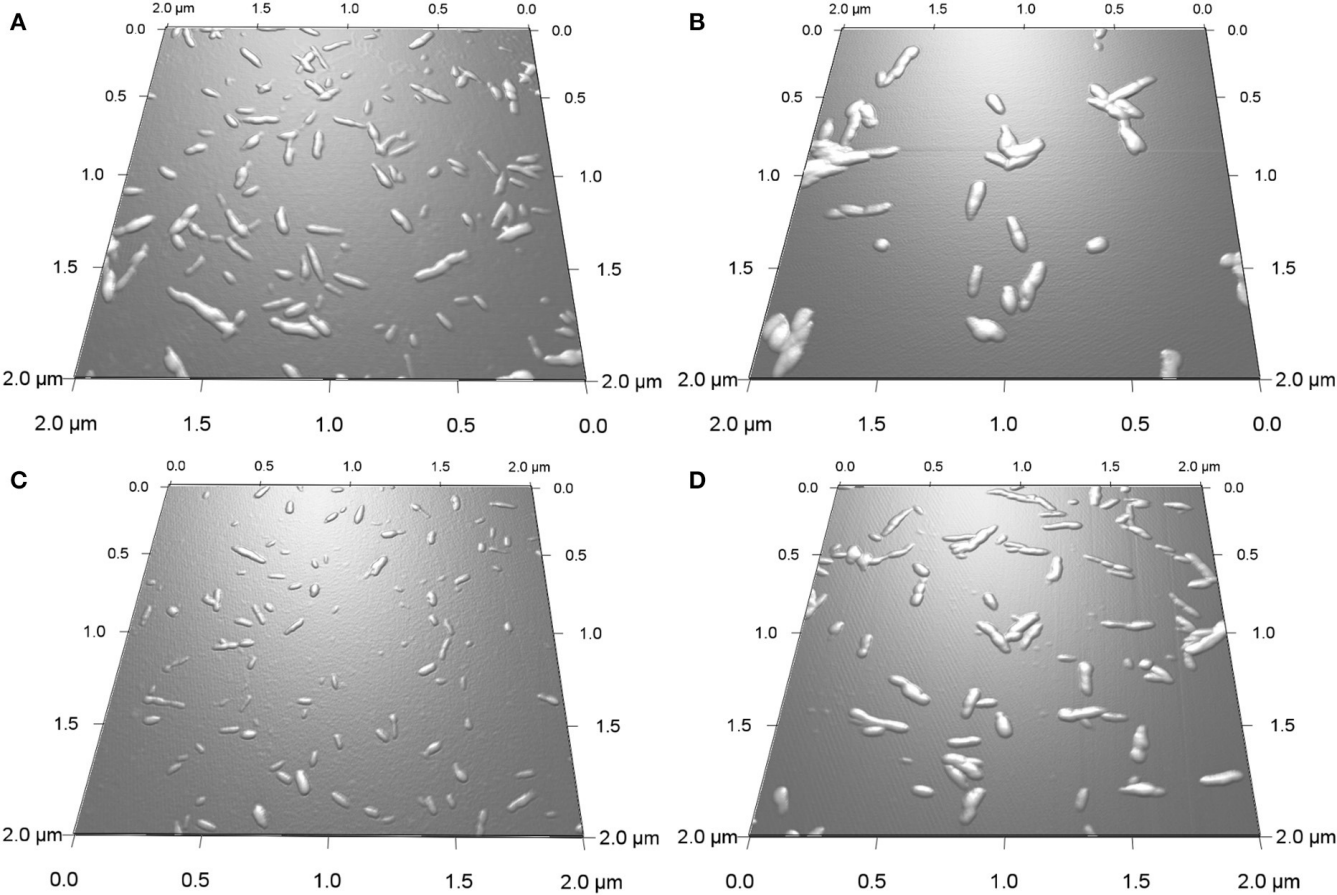
3D AFM height images of SH-CNCs **(A,B)** and SO-CNCs **(C,D)** before **(A,C)** and after **(B,D)** 400 h incubation in SBF at 37°C.

The effect of the type of surface group, sulfate vs. carboxyl, on the *in vitro* mineralization of CNCs was evaluated by incubation of the CNCs in SBF (1x). Figure 2 shows 3D AFM height images of CNCs before (0 h) and after incubation at 37°C for 400 h. Both CNCs appeared significantly larger after mineralization. The mean particle dimensions were determined from AFM height images. Before mineralization, SH-CNCs and SO-CNCs had a mean length of 121.5 ± 23.8 nm and a mean height of 3.86 ± 1.1 nm in accordance with the literature (Moon et al., 2011). The initial dimensions of SO-CNCs (mean length of 89.3 ± 22.7 nm and mean height of 2.23 ± 0.6 nm) were smaller, indicating an effect of the oxidation procedure on particle size. After 400 h of incubation in SBF, the size of CNCs was noticeably larger (Figures 2B,D) than before (Figures 2A,C). While the CNCs did not aggregate before incubation because of their negative surface charge (Figures 2A,C), mineralized CNCs exhibited some degree of aggregation (Figures 2B,D), possibly indicating a screening of their surface charge. Sonication was not performed during sample preparation for AFM analysis because it could affect the thickness of the mineral layer on the mineralized CNCs. For error minimization, only individual nanoparticles were used for height determination.

**Figure 3:**
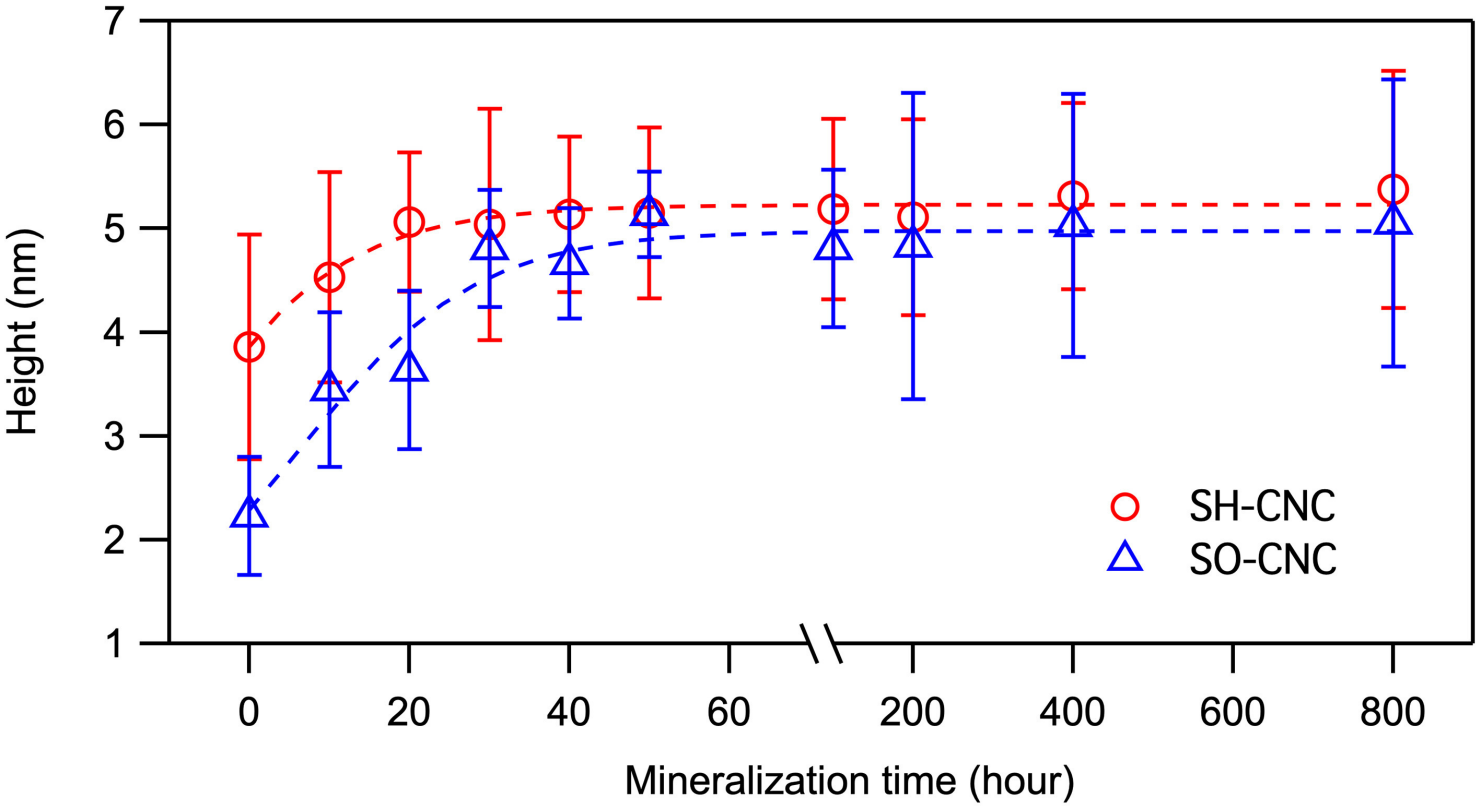
Height changes of CNCs during the incubation in SBF at 37°C.

The extent of mineralization was quantified using height data because width and length data of CNCs have greater natural fluctuations. Moreover, as opposed to length and width measurements, AFM height measurements are not affected by tip broadening and therefore generally considered more accurate. The average height of the CNCs as a function of incubation time is shown in Figure 3. The initial mean height of SO-CNCs was smaller than that of SH-CNCs because of the effect of the oxidation process on particle size. For the SH-CNCs, the height was significantly increased from 0 h (3.9 ± 1.1 nm) to 10 h (4.5 ± 1.0 nm) and from 10 h to 40 h (5.1 ± 0.7 nm), but no statistical difference (Least Significant Difference (LSD) test at the 0.05 level) was observed from 20 h (5.1 ± 0.7 nm) to 800 h (5.4 ± 1.1 nm). For the SO-CNCs, the height was increased from 0 h (2.2 ± 0.6 nm) to 10 h (3.4 ± 0.7 nm) and from 20 h (3.6 ± 0.8 nm) to 30 h (4.8 ± 0.6 nm), but there was no statistically significant difference between 30 and 800 h (5.1 ± 1.4 nm). Although a similar trend for the height change was observed for both CNCs, the total increase in height was greater for the SO-CNCs (2.3 times) than for the SH-CNCs (1.4 times). Considering the rod-like shape of CNCs, the height increase indicates that the SO-CNCs exhibited a thicker coating (~1.45 nm thickness) with certain minerals than the SH-CNCs (~0.75 nm thickness).

For the SH-CNCs, the height was significantly increased from 0 h (3.9 ± 1.1 nm) to 10 h (4.5 ± 1.0 nm) and from 10 to 40 h (5.1 ± 0.7 nm), but no statistical difference (Least Significant Difference (LSD) test at the 0.05 level) was observed from 20 h (5.1 ± 0.7 nm) to 800 h (5.4 ± 1.1 nm). For the SO-CNCs, the height was increased from 0 h (2.2 ± 0.6 nm) to 10 h (3.4 ± 0.7 nm) and from 20 h (3.6 ± 0.8 nm) to 30 h (4.8 ± 0.6 nm), but there was no statistically significant difference between 30 and 800 h (5.1 ± 1.4 nm). Although a similar trend for the height change was observed for both CNCs, the total increase in height was greater for the SO-CNCs (2.3 times) than for the SH-CNCs (1.4 times).

**Figure 4:**
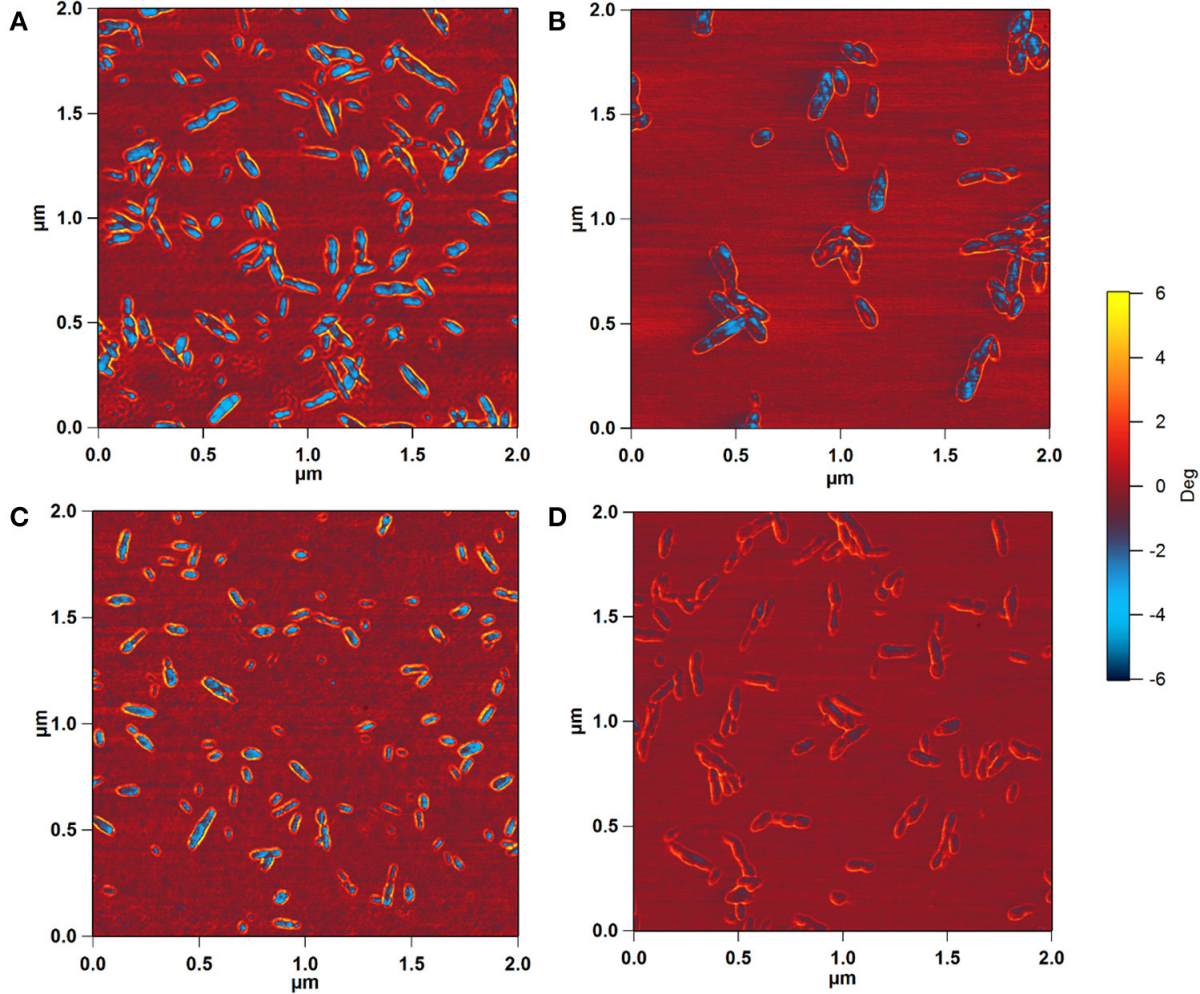
AFM phase images of SH-CNCs **(A,B)** and SO-CNCs **(C,D)** before **(A,C)** and after **(B,D)** 400 h incubation in SBF at 37°C. Scan size: 2 μm x 2 μm.

To determine whether the observed height increase was due to aggregation of CNCs, the AFM phase images, collected together with the height images, were analyzed. AFM intermittent contact mode provides information about sample topography and surface properties, such as stiffness, viscoelasticity, or surface energy, by monitoring the phase shift (also known as phase lag) of the cantilever oscillation relative to the drive signal (Stark et al., 2001; Garcia et al., 2006, 2007) during a scan. The phase shift allows identification of regions of different tip-sample interactions through the amount of energy dissipated and thus visualization of different surface charge characteristics (Tamayo and Garcia, 1997; Cleveland et al., 1998; Czajkowsky et al., 1998). Figure 4 shows the phase images corresponding to the height images in Figure 2. The phase shift of the substrate was about ±0.6°. For pristine SH-CNCs and SO-CNSs, the phase shift caused by the interactions between the CNCs and the AFM tip was 8.17° ± 1.11 (Figure 4A) and 7.88° ± 0.98 (Figure 4C), respectively, and there was no statistically significant difference (LSD test at the 0.05 level) between them. However, upon incubation in SBF, the phase shift was significantly reduced to 5.26° ± 0.81 (Figure 4B) in the case of SH-CNCs and 2.78° ± 0.73 in the case of SO-CNCs (Figure 4D). Furthermore, the decreases in phase shift for SH-CNCs and SO-CNCs of 2.91 ± 1.37° and 5.10 ± 1.22°, respectively, were statistically significantly different from one another.

The elemental composition of the surface deposit was analyzed by ICP AES. The elements that are of most interest for bone tissue engineering applications are calcium and phosphorus, making up half of the mass of the bone mineral HA. Table 2 compares the calcium and phosphorus concentrations and Ca:P ratios measured for the two CNC samples with those obtained for a synthetic HA (≥97%, Sigma-Aldrich), having a composition of Ca_10_(PO_4_)_6_(OH)_2_ and a Ca:P ratio of 2.15 by atomic weight and 1.67 by atomic ratio. The Ca:P ratios obtained for the mineralized SH-CNCs and SO-CNCs differed substantially from the Ca:P ratio of the HA control. Specifically, the Ca:P ratios for the CNCs were significantly higher than that of the control. Attempts to detect and identify the calcium phosphate mineral on the surface of the CNCs by X-ray diffraction were unsuccessful (Supplementary Figure 5).

**Table 2:** ICP AES analysis of CNCs before and after mineralization.

		**Ca (mg/L)**		**P (mg/L)**		**Ca:P**	
**Sample**	**Time (h)**	**Mean**	**STDEV**	**Mean**	**STDEV**	**Mean**	**STDEV**
SH-CNC	0	<0.021	–	<0.015	0.001	–	–
	50	0.387	0.002	0.147	0.002	2.64	0.029
	800	0.292	0.001	0.035	0.001	8.25	0.327
SO-CNC	0	0.101	0.001	<0.015	–	–	–
	50	4.61	0.025	0.118	0.004	39.0	1.40
	800	1.99	0.017	0.060	0.005	33.4	2.82
HA	–	147.0	1.93	68.5	0.132	2.15	0.028
2% HNO_3_	–	<0.021	–	<0.015	–	–	–

*Samples were prepared in aqueous 2% HNO3, Data reported as mg/L, The “<” indicates concentrations less than the instrument detection limit. Time (h) indicates the incubation period at 37°C for the mineralization*.

**Figure 5:**
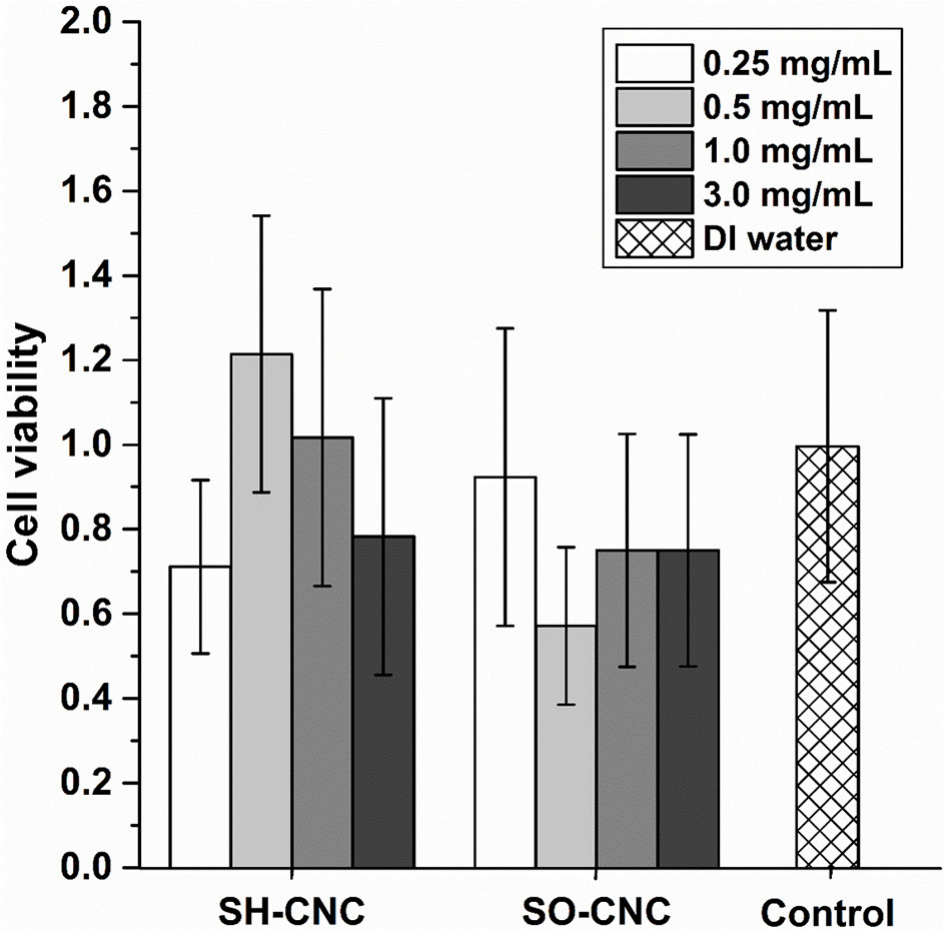
Cytotoxicity of MC3T3 cells after 24 h exposure to SH-CNCs and SO-CNCs. Each data set mean value was normalized to the DI water control, data showed no significant difference in means (*p* < 0.05) compared to the control. Error bars represent one standard deviation.

The toxicity of SH-CNCs and SO-CNCs to MC3T3 preosteoblasts was analyzed with a trypan blue viability assay (Figure 5). Suspensions of SH-CNCs and SO-CNCs in DI water were added to MC3T3 cells in culture medium for final concentrations of 0.25, 0.5, 1.0, and 3.0 mg/mL. Dynamic light scattering analysis of SH-CNCs and SO-CNCs in culture medium (Supplementary Figure 6 and Supplementary Table 2) indicated that SO-CNCs maintained a monomodal size distribution with a z-average ranging from 100 to 160 nm, depending on the concentration, whereas SH-CNCs exhibited a multi-modal size distribution with z-average values as large as 2 μm, indicating significant particle aggregation. There was no statistically significant difference in cell viability of MC3T3 cells exposed to SH-CNCs or SO-CNCs compared to cells exposed to pure DI water (control, Figure 5). Thus, both SH-CNCs and SO-CNCs were non-toxic to MC3T3 cells at concentrations of up to 3 mg/mL during 24 h of exposure.

Bone scaffolds for critical-sized defects need to have adequate mechanical properties. Here, the mechanical properties of SO-CNC/PCL nanocomposites were evaluated as a function of SO-CNC content. A general challenge in the development of polymer-based nanocomposites with naturally derived nanoparticles is achieving homogenous dispersion of the nanoparticles in the polymer matrix. Most naturally derived nanoparticles, such as polysaccharide-based nanoparticles, have hydrophilic groups (e.g., hydroxyl, carboxyl, and amino groups) whereas bioresorbable polymers are relatively hydrophobic. Inhomogeneous nanoparticle distribution can lead to undesirable properties and phase separation. Several methods have been reported for improving the dispersion of CNCs in polymer matrices. For example, CNCs were transferred from an aqueous to a non-aqueous system (e.g., organic media) prior to incorporation into the polymer matrix (Samir et al., 2004; Kvien et al., 2005; Marcovich et al., 2006; van den Berg et al., 2007) or chemically modified with hydrophobic functional groups (Grunert and Winter, 2002; Gousse et al., 2004), grafting-onto (Araki et al., 2001; Habibi and Dufresne, 2008), and grafting-from (Habibi et al., 2008) approaches to increase the compatibility.

In the present study, we aimed to prevent the aggregation of SO-CNCs in the PCL matrix with minimal use of harmful organic solvents through a simple four-step process including (a) solvent exchange of an aqueous SO-CNC suspension, (b) physical mixing, (c) solvent casting, and (d) melt compounding extrusion. First, the SO-CNCs were dispersed in CH_3_CN by solvent exchange and the PCL pellets were dissolved in CH_2_Cl_2_ which is miscible with CH_3_CN. After that, the dissolved PCL was slowly added to the SO-CNCs suspended in CH_3_CN under stirring in ultrasonic bath. The SO-CNC/PCL nanocomposite filaments were prepared by solvent casting, followed by melt compounding extrusion using a twin-screw extruder.

**Figure 6:**
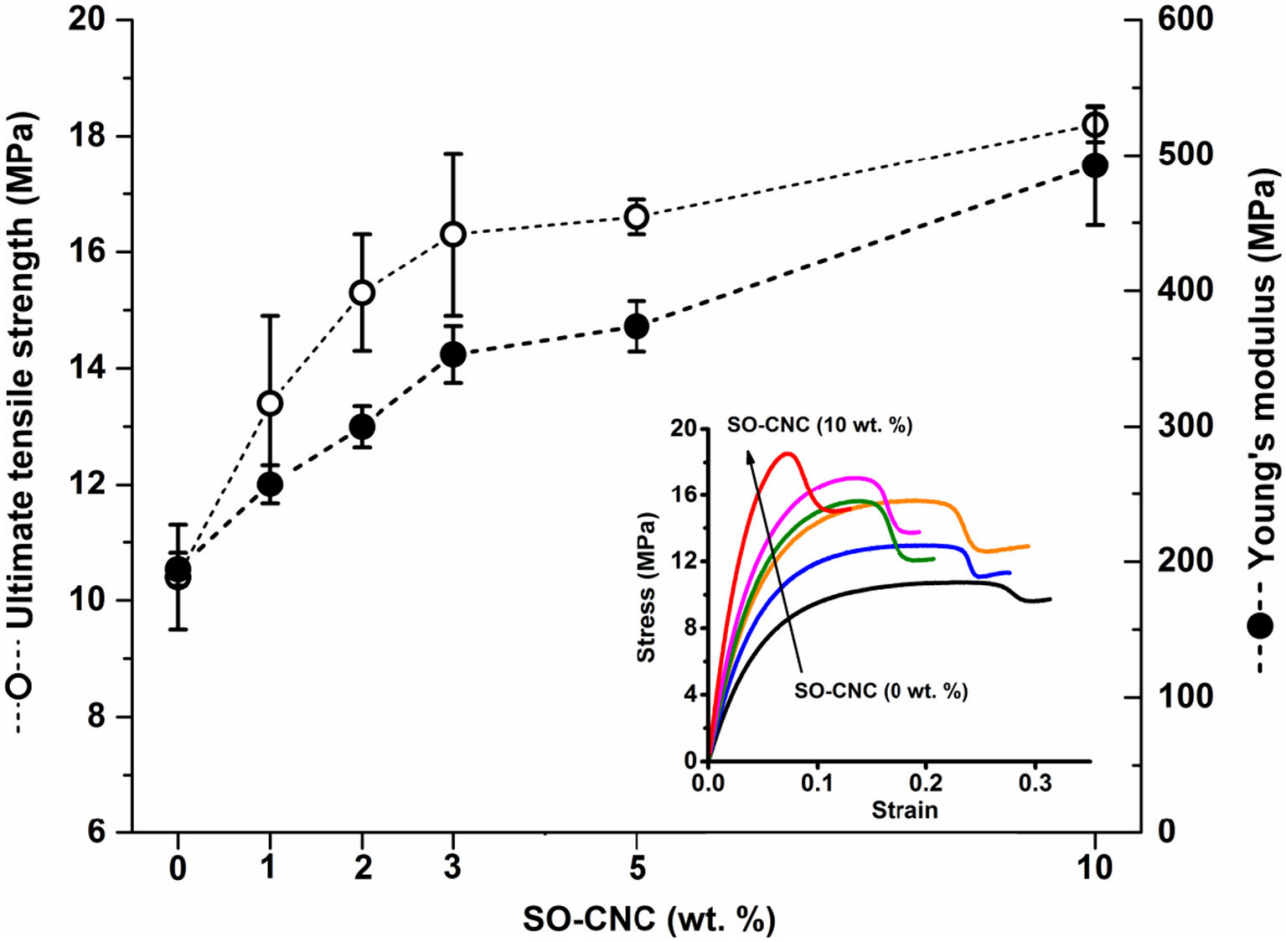
Mechanical properties of SO-CNC/PCL nanocomposite filaments. Insert: Stress-strain curves of the nanocomposite filaments.

The mechanical properties of the SO-CNC/PCL nanocomposite filaments were determined by tensile testing. Figure 6 shows the tensile strength and Young's modulus as a function of SO-CNC content. Both, a decrease in ductility and pronounced mechanical reinforcement were observed in the stress-strain curves with increasing SO-CNC content. The maximum values of both strength (18.2 ± 0.3 MPa) and Young's modulus (492.5 ± 44.1 MPa) were obtained at 10 wt% loading of SO-CNCs. These mechanical properties are very similar to those of human cancellous bone [tibia, tension test, strength: 2.54 ± 1.18 MPa, Young's modulus: 483 MPa (Rohl et al., 1991)].

Attempts to assess the degree of dispersion of the SO-CNCs in the PCL matrix were moderately successful. Supplementary Figure 7 shows optical microscopy images of the SO-CNC/PCL nanocomposites after heating to 300°C for 10 min. The high temperature results in thermal degradation of the SO-CNCs (Supplementary Figure 2), rendering them visible by discoloration. The discoloration was relatively uniform throughout the nanocomposites, although some larger dark patches indicated a certain degree of SO-CNC aggregation in the PCL matrix, which was more pronounced at higher SO-CNC contents. AFM phase images of the SO-CNC/PCL nanocomposites (Supplementary Figure 8) also confirmed an overall uniform distribution of SO-CNCs with regions of higher local concentration on a smaller scale and a more pronounced effect at higher SO-CNC contents.

**Figure 7:**
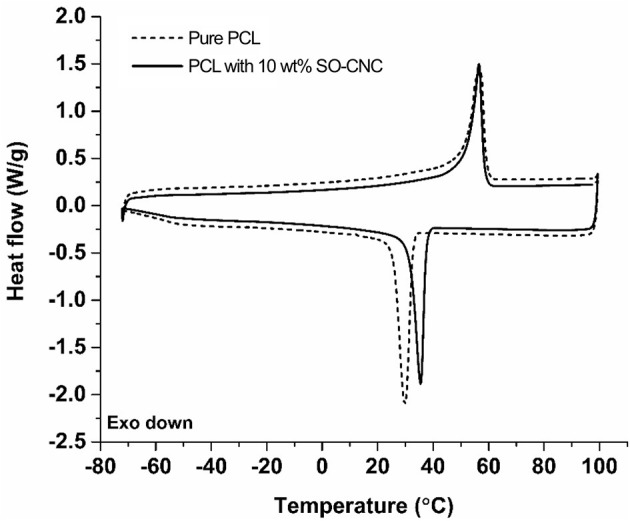
DSC curves (2nd heating and 2nd cooling) of pure PCL and SO-CNC/PCL nanocomposite filament containing 10 wt% SO-CNC.

The thermal properties of the nanocomposites were evaluated by DSC. The 1st heating cycle, from −75°C to 100°C, eliminated the thermal history during the nanocomposite fabrications. Identical DSC curves were observed for the 1st cooling and 2nd cooling cycles. The 2nd heating and cooling curves of pure PCL and PCL with 10 wt% SO-CNCs are shown in Figure 7. The thermal transitions are summarized in Table 3. The enthalpy of fusion, Δ*H*_*f*_, was measured from the 2nd heating cycles (area under the melting peak) to calculate the percent crystallinity (*X*_*c*_). The glass transition (*T*_g_: ~ −64°C) and melting (*T*_m_: ~56°C) temperatures were not influenced by the addition of SO-CNCs. However, the crystallization temperature (*T*_c_) of the nanocomposites increased by about 4.5°C upon addition of 1 wt% SO-CNCs and by another ~1°C upon increase of the SO-CNC content to 10 wt%. The calculated Δ*H*_*f*_ gradually decreased from about 60 J/g (0 wt% SO-CNC) to about 50 J/g (10 wt% SO-CNC) and the crystallinity of the nanocomposites decreased from about 43% to about 40% with increasing SO-CNC content (from 0 to 10 wt%).

**Table 3:** Summary of mechanical properties, thermal transitions, crystallinity, and contact angle measurements of the SO-CNC/PCL nanocomposites.

**SO-CNC content (wt%)**	**Mechanical properties**		**Thermal transitions**			**Crystallinity**	**Contact angle**	
	**Ultimate strength (MPa)**	**Young's modulus (MPa)**	**T_g_ (°C)**	**T_c_ (°C)**	**T_m_ (°C)**	**X_c_ (%)**	**DI water (°)**	**SBF (°)**
0	10.4 ± 0.9	194.3 ± 12.1	−64.6 ± 0.6	30.2 ± 0.3	56.0 ± 0.6	42.9 ± 0.7	87.0 ± 1.1	88.8 ± 0.9
1	13.4 ± 1.5	257.2 ± 14.4	−64.9 ± 0.8	34.6 ± 0.2	56.1 ± 0.1	42.5 ± 0.4	85.5 ± 0.9	88.7 ± 0.6
2	15.3 ± 1.0	299.9 ± 15.2	−64.7 ± 0.5	34.5 ± 0.1	56.2 ± 0.1	42.1 ± 0.6	–	–
3	16.3 ± 1.4	353.1 ± 20.9	−64.4 ± 0.9	35.5 ± 0.1	56.1 ± 0.1	40.5 ± 0.6	84.5 ± 1.2	85.4 ± 1.1
5	16.6 ± 0.3	373.8 ± 18.6	−64.6 ± 0.8	35.2 ± 0.3	56.4 ± 0.2	39.9 ± 0.9	83.6 ± 1.4	85.2 ± 0.7
10	18.2 ± 0.3	492.5 ± 44.1	−64.5 ± 0.4	35.7 ± 0.2	56.4 ± 0.4	39.7 ± 0.5	82.6 ± 1.9	82.6 ± 0.5

*Tg, glass transition temperature; Tc, crystallization temperature; Tm, melting temperature*.

*Xc calculated from 1st heating cycle: 49.6 ± 0.6% (SO-CNC content: 0 wt%), 50.0 ± 1.0% (1 wt%), 50.6 ± 1.0% (2 wt%), 49.3 ± 0.6% (3 wt%), 49.0 ± 0.6% (5 wt%), and 48.9 ± 1.1% (10 wt%)*.

It is essential for implant materials to achieve an appropriate cellular response for tissue regeneration. The material surfaces will have direct contact with the biological environment and the resulting responses are critically important for successful implantation in tissue engineering applications. For bone scaffold materials, for example, the interactions between cell and material can promote preosteoblastic cell attachment, migration, proliferation, differentiation, and bioactivity (bone formation) (Wilson et al., 2005). It is commonly observed that hydrophilic surfaces provide a better environment for cell adhesion, but many bioresorbable polymers, including PCL, are relatively hydrophobic. The effect of the hydrophilic SO-CNCs on the PCL hydrophobicity was assessed by contact angle measurements. The average values obtained for DI water and SBF on SO-CNC/PCL nanocomposites are listed in Table 3. The contact angles of DI water and SBF decreased from 87.0 ± 1.1° and 88.8 ± 0.9° on pure PCL to 82.6 ± 1.9° and 82.6 ± 0.5° on the SO-CNC/PCL nanocomposite with 10 wt% SO-CNCs, respectively. This decrease in contact angle indicates an overall decrease in PCL hydrophobicity upon addition of the hydrophilic SO-CNCs.

## Discussion

The surface chemistry of SH-CNCs is dominated by sulfate half ester groups introduced during the cellulose hydrolysis with sulfuric acid. TEMPO-mediated oxidation of SH-CNCs, to produce SO-CNCs, selectively oxidizes the primary hydroxyl groups of cellulose. The obtained surface charge density of SO-CNC-I of 1.997 ± 0.142 mmol/g suggests that the reaction resulted in the conversion of approximately one third of the available surface hydroxyl groups, not carrying sulfate half-esters, to carboxyl groups. The 21% reduction in sulfur content upon surface oxidation indicates that SO-CNCs still contain about 80% of the initial sulfate groups. The high surface charge density of 1.997 ± 0.142 mmol/g signifies, however, that carboxyl groups are the primary functional group on SO-CNCs, accounting for 86–89% of the anionic surface groups.

Kokubo and Takadama (2006) reviewed correlations between apatite formation on the surface of various materials in SBF *in vitro* and their *in vivo* bioactivity. The researchers concluded that there is no correlation between apatite formation in SBF at non-natural concentration levels (e.g., 1.5x SBF) and its *in vivo* formation on a material. In contrast, results obtained using 1x SBF correlated well with *in vivo* bioactivity. This observation is crucial for the successful development of new bioactive bone scaffold materials on the basis of predictions from *in vitro* apatite formation on the material's surface. Although numerous studies using SBF with several-fold differences in concentration have reported *in vitro* HA formation with a Ca:P ratio of ~1.67 on a bone scaffold material, it must be pointed out that the use of such fluids does not allow conclusions with regard to the ability of these materials to promote biomineralization *in vivo*.

In light of the observation of Kokubo and Takadama, the biomineralization experiments in this study were carried out with 1x SBF. Upon incubation in SBF, SO-CNCs exhibited a 64% greater height increase than SH-CNCs. The greater total height increase indicates that SO-CNCs build a thicker mineral coating (~1.45 nm thickness) than the SH-CNCs (~0.75 nm thickness) when exposed to calcium and phosphate ions. The thicker coating of SO-CNCs could be due to their higher surface charge density or the higher calcium affinity of the carboxyl group, compared to the sulfate group.

Prior to incubation in SBF, both SH-CNCs and SO-CNCs showed stronger interactions with the AFM tip compared to the mica substrate, as indicated by the greater phase shift. Upon incubation in SBF, the phase shift of both SH-CNCs and SO-CNCs was significantly reduced and SO-CNCs showed a statistically significantly greater decrease in phase shift relative to SH-CNCs. The mica substrate, which is mainly composed of SiO_2_ (~46%) and Al_2_O_3_ (~33%), exhibited a very small phase shift, indicative of a hydrophobic surface (Boussu et al., 2005). The decrease in phase shift upon incubation in SBF signifies a decrease in the surface hydrophilicity of SH-CNCs and SO-CNCs, suggesting that the height increase was not caused by particle aggregation but by a buildup of a more hydrophobic mineral coating. The greater decrease observed with the SO-CNCs compared to the SH-CNCs could be due to a thicker mineral coating or a difference in mineral composition, resulting in a more hydrophobic composition on the SO-CNCs.

Analysis of the elemental composition of the mineral coating yielded much higher Ca:P ratios than expected for bone-like apatite or HA and an apparent absence of mineral diffraction peaks in X-ray diffractograms. The mechanism of HA formation is highly complex and still under investigation. Dey et al. (2010) demonstrated that the development of oriented apatite crystals is induced by the densification of amorphous calcium phosphate (ACP) at a templating surface, prior to which calcium phosphate prenucleation clusters (0.87 ± 0.2 nm in diameter) aggregate in equilibrium with ions in solution (SBF). In our study, the negatively charged CNCs, suspended in SBF, may have hindered the formation of prenucleation clusters by binding to and immobilizing SBF cations. The experimental setup in this study differed from those in other studies where the substrates were secured (commonly at the bottom) during the experiment. Consequently, the significantly higher Ca:P ratios in this study could be due to binding of calcium ions by the negatively charged surface groups of the CNCs suspended in the SBF.

Similar results have been reported by Zurick et al. (2013), who investigated the mineralization induction capabilities of the primary non-collagenous proteins bone sialoprotein (BSP), osteopontin (OPN), and the calcium binding subdomain of dentin sialophosphoprotein, dentin phosphoprotein (DPP). All minerals formed under the conditions used in this investigation had Ca:P ratios that were significantly larger than what has been found in native bone tissue. Proteins containing the carboxy-terminal fragment are highly negatively charged and have calcium chelating properties. The significantly larger Ca:P ratios observed (~4–20) indicate a potential effect of the terminal carboxyl groups in mineralization process (Prasad et al., 2010; Zurick et al., 2013). One should, however, keep in mind that the mineralization of bone (or bone formation) only occurs if bone-forming cells (osteoblasts) and other biological factors are intimately involved and regulated properly in the body, which is a very complex process to mimic.

The greater bioactivity of SO-CNCs with respect to biomineralization in SBF, compared to SH-CNCs, combined with an equal lack of cytotoxicity, suggests that SO-CNCs are superior candidates for bone tissue engineering applications than SH-CNCs.

SO-CNCs proved highly effective as reinforcing nanofillers in PCL-based nanocomposites. An SO-CNC content of 10 wt% resulted in a more than 2-fold increase in Young's modulus (stiffness) and a more than 60% increase in ultimate tensile strength. The observed crystallinity decrease is in agreement with the literature. A decrease in the degree of crystallinity is commonly observed in the presence of particulates because particulates may act as nucleating agents and the filler-polymer interfaces provides heterogeneous nucleating sites (Hikosaka et al., 2006). The restricted mobility of polymer chains near the filler-polymer interface hinders the development of defect-free lamellar crystals and restricts the diffusion of polymer chains, thus affecting crystallization rate (Di Maio et al., 2004). The increase in crystallization temperature suggests that the SO-CNCs acted as nucleation sites for PCL crystallization and the decreased crystallinity in the SO-CNC/PCL nanocomposites, compared to pure PCL, could be a result of increased restriction of polymer mobility and diffusion near the SO-CNC surface, resulting in smaller PCL crystals with a greater number of lattice defects.

The decrease in hydrophobicity with increasing SO-CNC content, demonstrated by the decrease in DI water and SBF contact angle, indicates a greater biocompatibility and bioactivity of the SO-CNC/PCL nanocomposites, compared to pure PCL. The effect of SO-CNC filler content on the proliferation and differentiation of MC3T3 propsteoblasts on 3D printed scaffolds will be reported separately.

## Summary

This study assessed the ability of SO-CNCs, with a carboxylate-governed surface chemistry, to induce biomineralization and enhance the mechanical properties of PCL for use in bone scaffolds. SO-CNCs showed a greater ability than SH-CNCs, with a sulfate-governed surface chemistry, to induce the deposition of a calcium-phosphate layer on the CNC surface. Neither SH-CNCs nor SO-CNCs were toxic to MC3T3 preosteoblasts during a 24 h exposure at concentrations ranging from 0.25 to 3.0 mg/mL. SO-CNCs significantly increased the ultimate tensile strength and Young's modulus of PCL at filler contents of up to 10 wt%. The SO-CNCs had no effect on PCL glass transition and melting temperature but increased the crystallization temperature and hydrophilicity of the nanocomposites. The effectiveness of SO-CNCs to enhance the mechanical properties of PCL and induce the deposition of a calcium-phosphate layer on their surface makes them interesting multi-functional additives for resorbable polymer-based bone scaffolds.

## Data Availability Statement

The raw data supporting the conclusions of this article will be made available by the authors, without undue reservation.

## Author Contributions

JH carried out all experiments except the cell culture experiments and drafted the article. SC carried out the cell culture experiments and provided information on the experimental details for these experiments. AW supervised the cell culture experiments and provided feedback on the article draft. MR guided the research and revised the article draft to the submitted version. All authors contributed to the article and approved the submitted version.

## Conflict of Interest

The authors declare that the research was conducted in the absence of any commercial or financial relationships that could be construed as a potential conflict of interest.
