# Supplementary material for: Bioactive Cellulose Nanocrystal-Poly(ε-Caprolactone) Nanocomposites for Bone Tissue Engineering Applications
Source: Front Bioeng Biotechnol. 2021 Feb 25;9:605924. doi: 10.3389/fbioe.2021.605924 (PMC7947866; doi:10.3389/fbioe.2021.605924)
Supplement: Supplementary file 1 [file Data_Sheet_1.PDF]

Supplementary Material

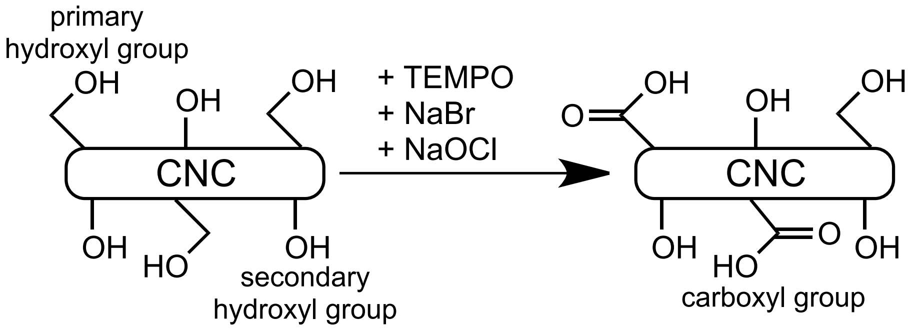

Supplementary Figure 1. Schematic illustration of the TEMPO-mediated oxidation of SH-CNCs. (Sulfate groups, present in both SH-CNC and SO-CNC, have been omitted for clarity.)

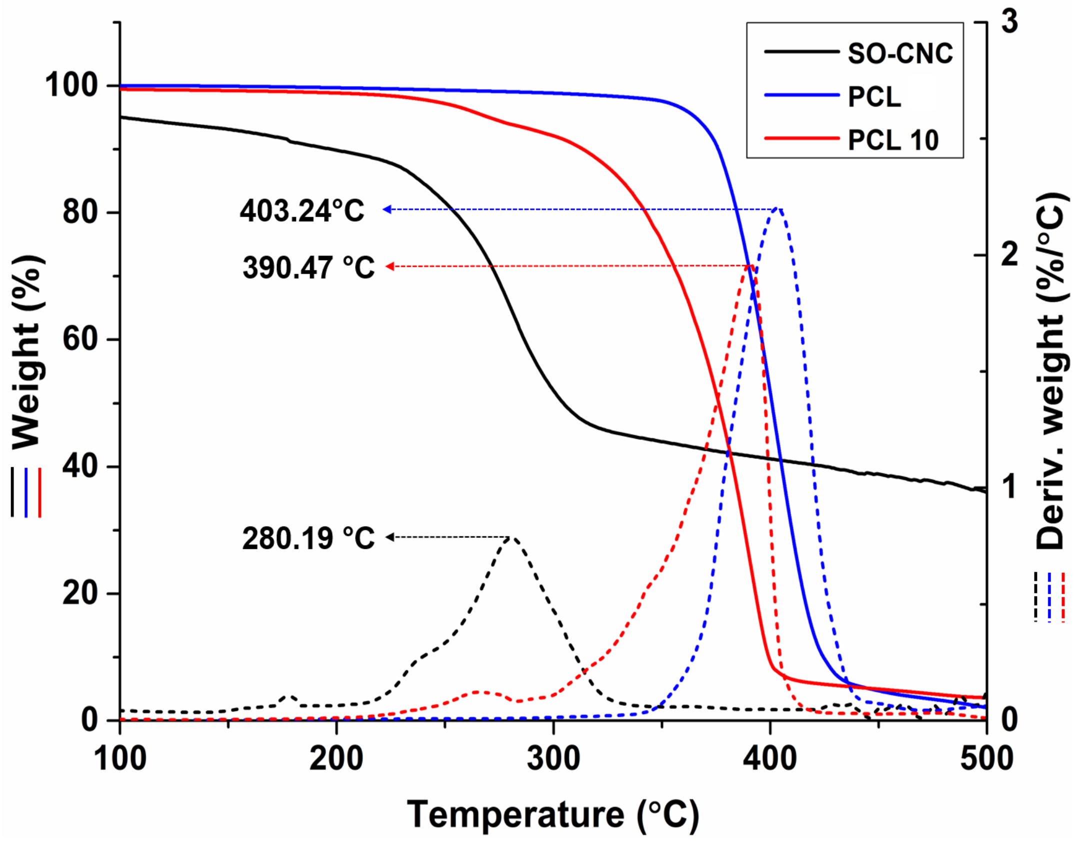

Supplementary Figure 2. TG (solid line) and DTG (dash line) curves for SO-CNC, pure PCL, and PCL with 10 wt% SO-CNCs ("PCL 10").

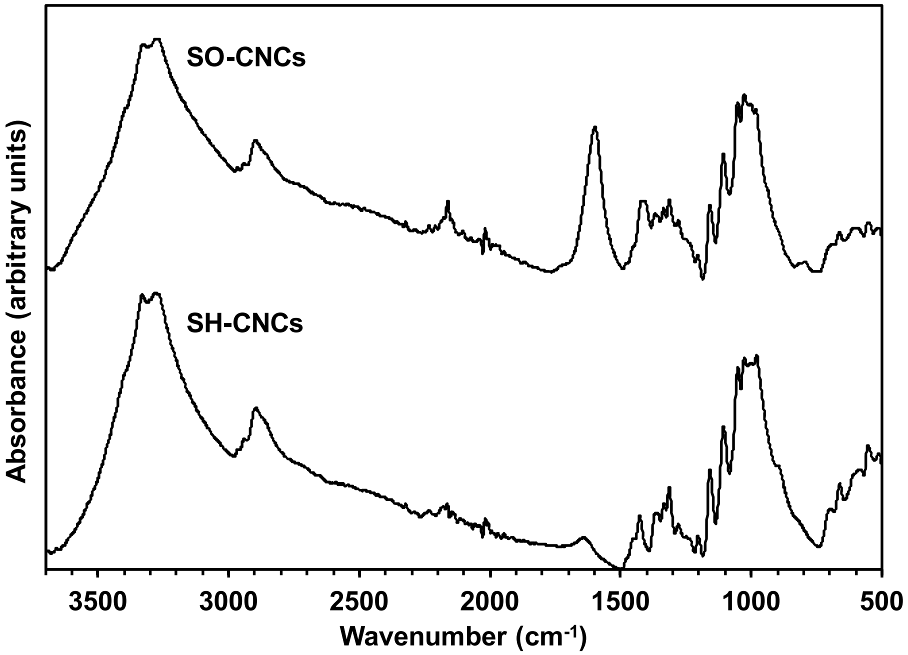

Supplementary Figure 3. FTIR spectra of SH-CNCs and SO-CNCs.

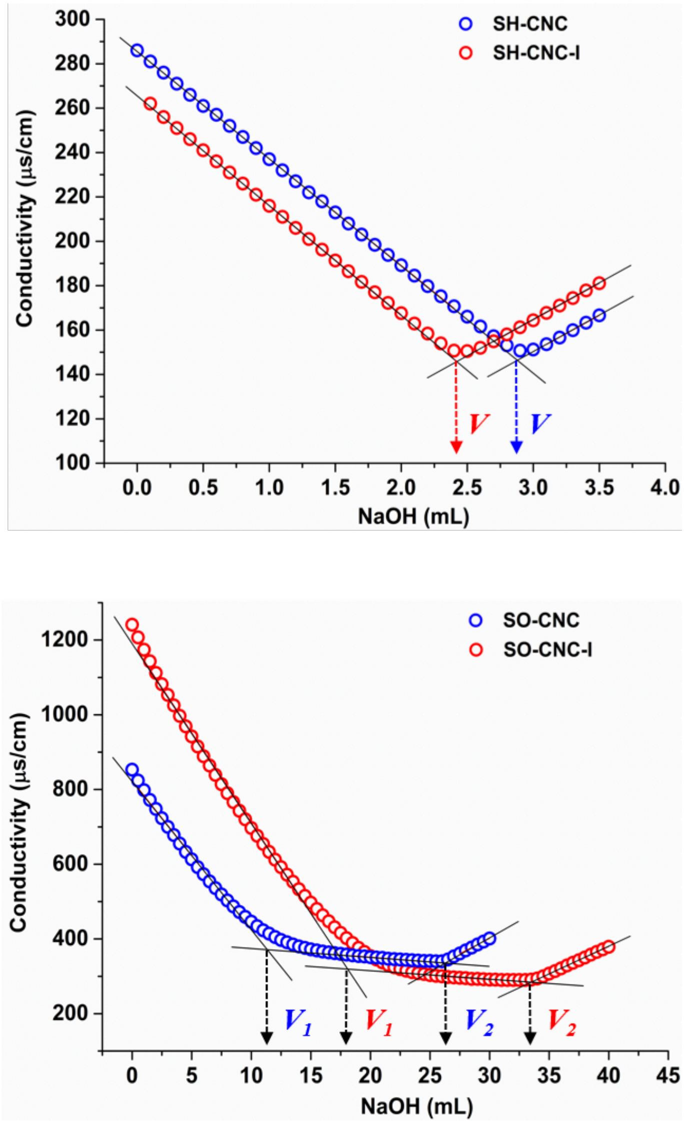

Supplementary Figure 4. Conductometric titrations of aqueous SH-CNC (top) and SO-CNC (bottom) suspension before (blue) and after ion exchange resin treatment for 12 h (red).

Supplementary Table 1. Summary of titration (surface charge density) and ICP analysis (sulfur and sodium) of CNCs before and after ion exchange resin treatment.

	Conductometric titration	ICP analysis	
	Surface charge density (mmol/g)	Analyte (mg/L)	
		S	Na
**SH-CNC**	0.316 ± 0.007	0.217 ± 0.04	0.020 ± 0.004
**SH-CNC-I**	0.271 ± 0.002	0.180 ± 0.004	0.052 ± 0.005
**SO-CNC**	1.840 ± 0.004	0.171 ± 0.004	0.881 ± 0.005
**SO-CNC-I**	1.997 ± 0.142	0.152 ± 0.002	0.065 ± 0.008

***** Surface charge density of SH-CNC/SH-CNC-I and SO-CNC/SO-CNC-I indicate sulfate group and carboxyl group, respectively.***

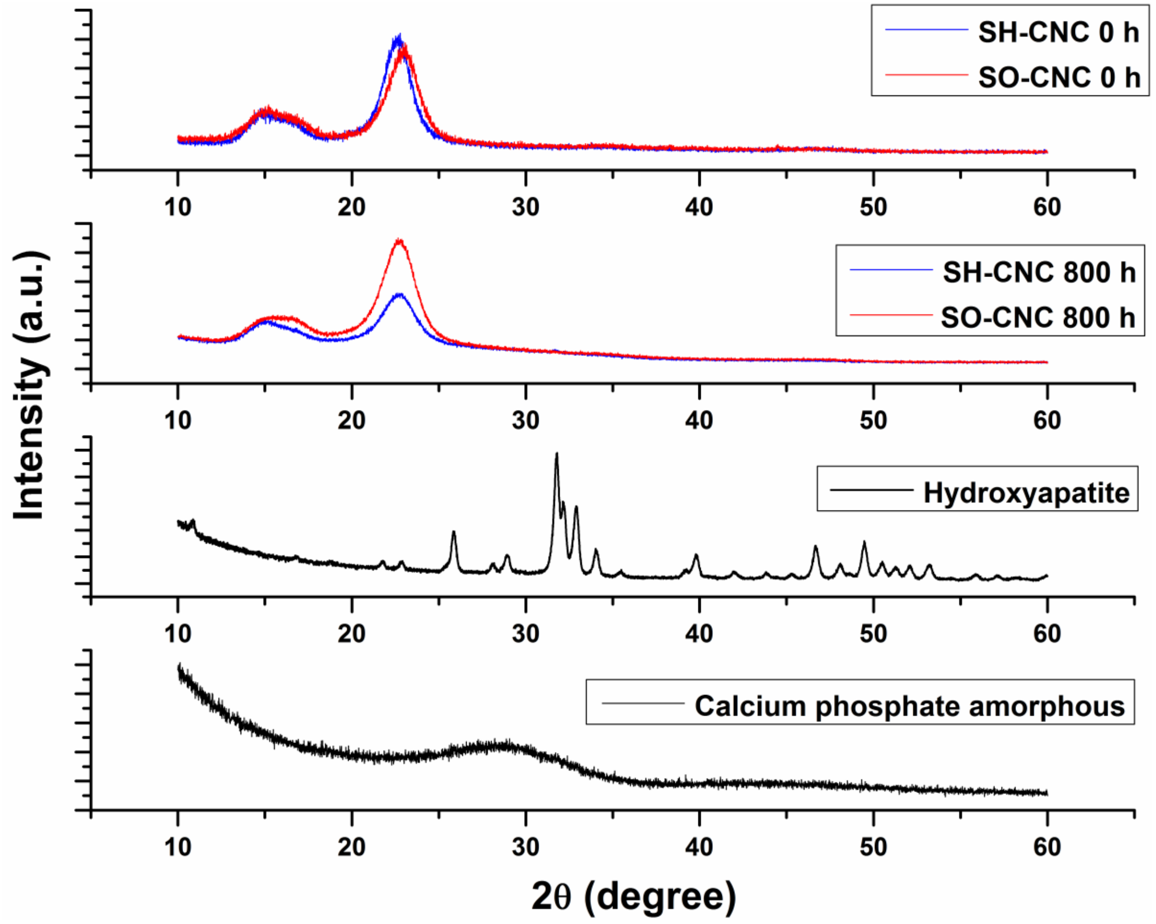

Supplementary Figure 5. X-ray diffraction pattern of CNCs before and after the mineralization compared to hydroxyapatite and calcium phosphate amorphous.

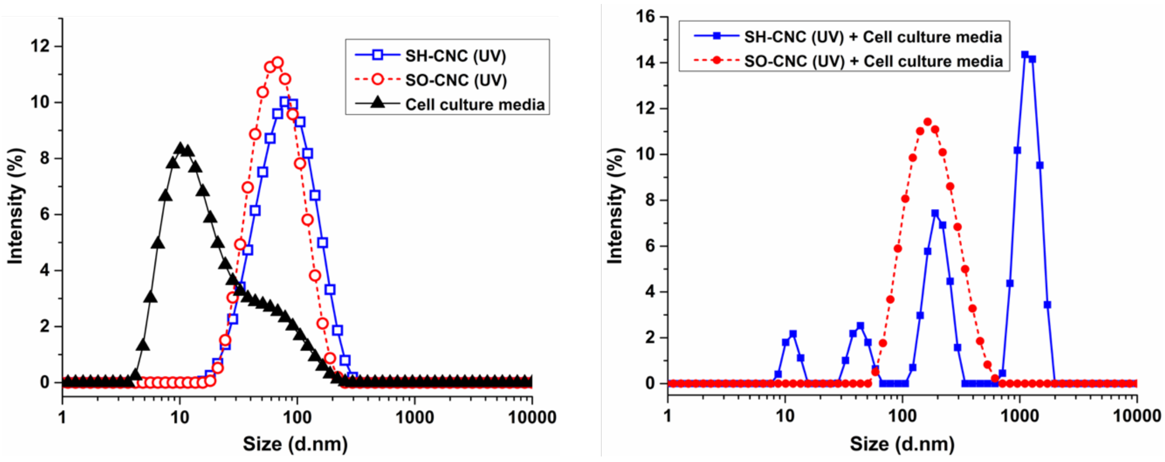

Supplementary Figure 6. Dynamic light scattering of CNCs in DI water after UV treatment (left), cell culture media only (left) and mixtures of CNCs and cell culture media (right) at 37 °C. The concentration of SH-CNC (pH=4.0) and SO-CNC (pH=6.3) in DI water and cell culture media was adjusted to 1 mg/mL.

Supplementary Table 2. Dynamic light scattering data for SH-CNCs and SO-CNCs in DI water and cell culture medium (α-MEM).

		DI water	DI water (UV)	DI water (UV) + Cell culture media			
	Concentration	1.0 mg/mL	1.0 mg/mL	0.25 mg/mL	0.5 mg/mL	1.0 mg/mL	3.0 mg/mL
SH-CNC	Z-average (d.nm)	64.2 ±0.2	68.5 ±3.1	1221.0 ±17.1	1669.3 ±37.2	1214.7 ±63.5	2018.0 ±1851.4
	PdI	0.19 ±0.01	0.23 ±0.00	0.97 ±0.05	1.0 ±0.00	0.98 ±0.02	0.82 ±0.31
SO-CNC	Z-average (d.nm)	52.0 ±1.60	55.0 ±2.02	157.7 ±6.7	143.9 ±9.0	143.4 ±5.4	99.7 ±6.2
	PdI	0.19 ±0.01	0.20 ±0.00	0.32 ±0.00	0.34 ±0.06	0.28 ±0.01	0.24 ±0.00

**** Nanoparticles in DI water before and after UV treatment were sonicated before measurement but the samples with cell culture media were not sonicated after mixing. The measurements were performed at 37 °C.***

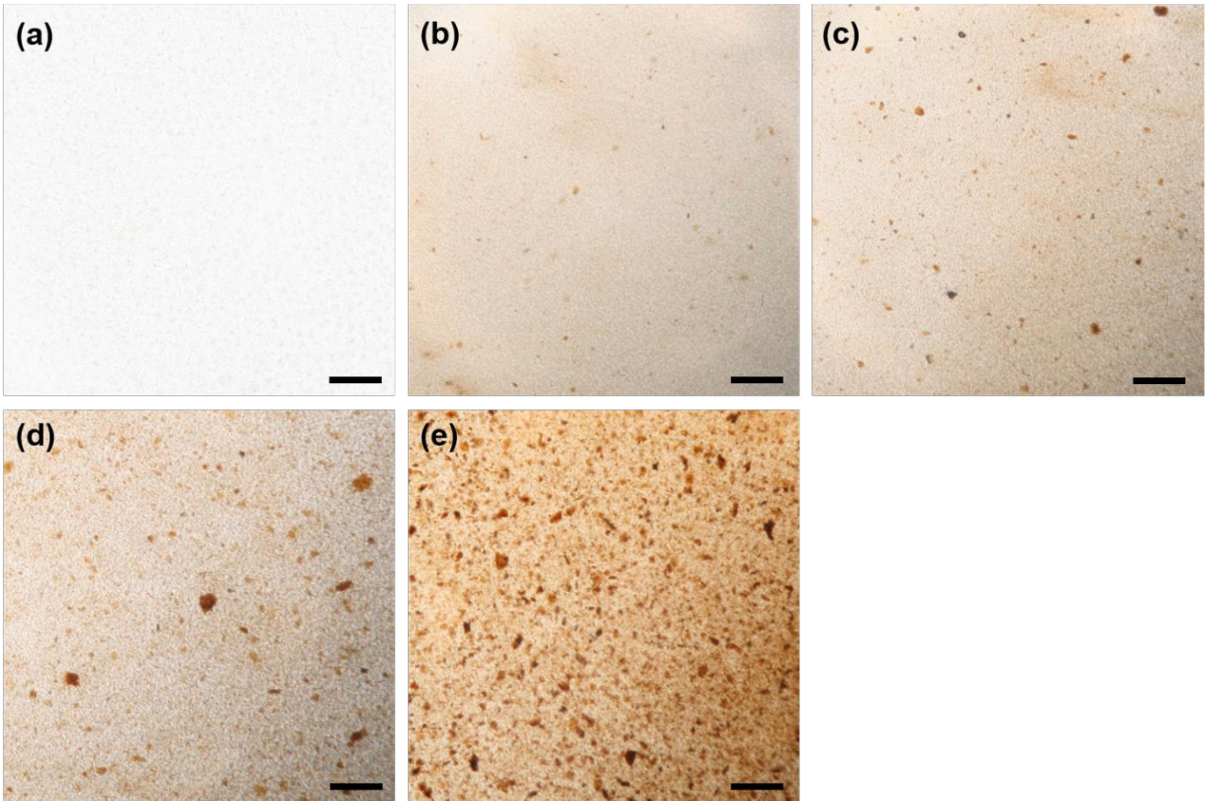

Supplementary Figure 7. Optical microscopy images of PCL nanocomposites with different SO-CNC contents, (a) 0 wt% (pure PCL), (b) 1 wt%, (c) 3 wt%, (d) 5 wt% and (e) 10 wt% after heating to 300 °C for 10 min.

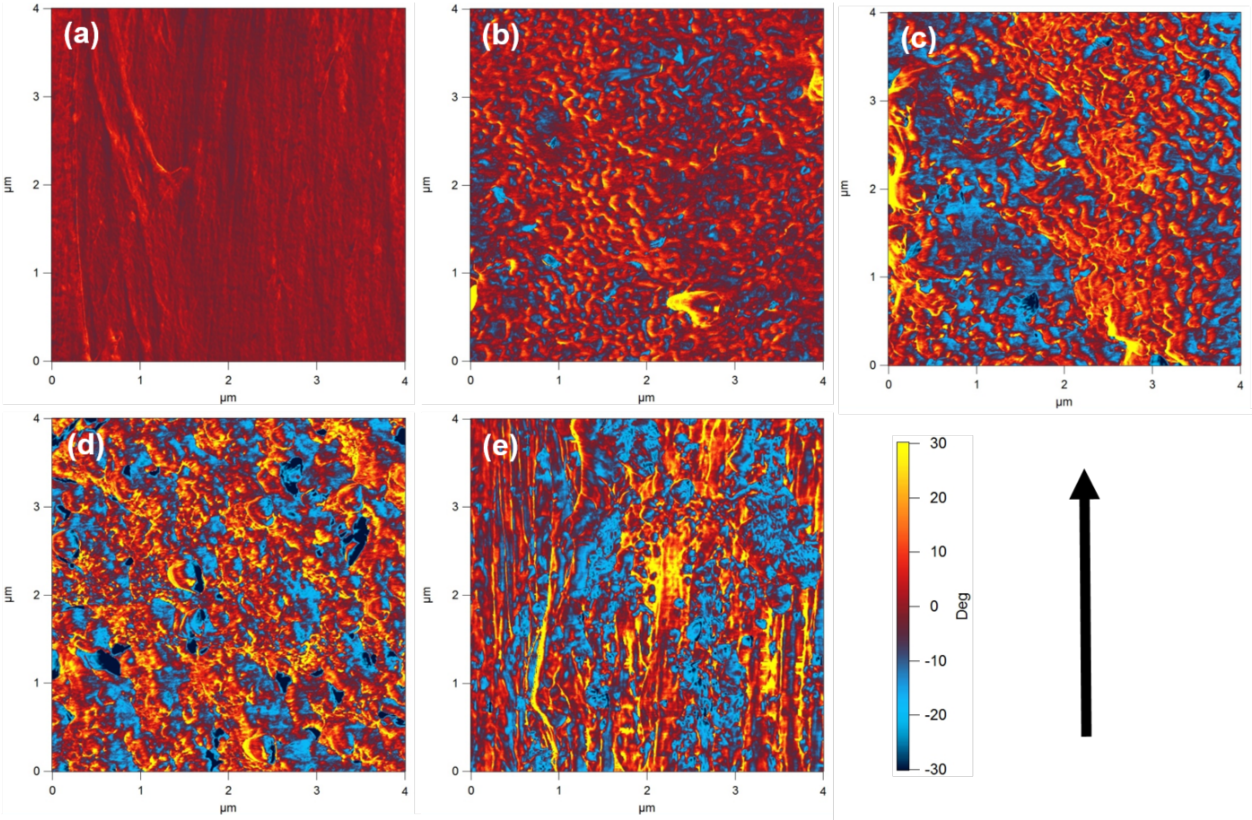

Supplementary Figure 8. AFM phase images of PCL nanocomposites with different SO-CNC contents, (a) 0 wt% (pure PCL), (b) 1 wt%, (c) 3 wt%, (d) 5 wt% and (e) 10 wt%. Scale bar represents the phase angle (°). Arrow indicates the flow direction during the melt compounding extrusion process.
